# A Case of Diverticular Perforation in a Young Patient with Rheumatoid Arthritis on Methotrexate

**DOI:** 10.1155/2015/617268

**Published:** 2015-04-29

**Authors:** Ian Chang, Carla Guggenheim, Heather Laird-Fick

**Affiliations:** ^1^Department of Medicine, Michigan State University, East Lansing, MI 48824, USA; ^2^EW Sparrow Hospital, Lansing, MI 48912, USA; ^3^Arthritis Care, PC, Lansing, MI 48906, USA

## Abstract

*Background*. Disease-modifying antirheumatic drugs (DMARDs), such as methotrexate (MTX), are associated with gastrointestinal toxicity. MTX inhibits dihydrofolate reductase, but it is unclear if polymorphisms of the methylenetetrahydrofolate reductase (*MTHFR*) gene predict toxicity. *Case*. We describe a 33-year-old male with polyarticular rheumatoid arthritis who developed sigmoid diverticular perforation while receiving methotrexate, folic acid, prednisone, and naproxen. He tested heterozygous for the C677T allele *MTHFR* gene. *Discussion*. Rheumatoid arthritis and its treatments are associated with increased risk of gastrointestinal disease. In one study, perforation was highest among individuals with concomitant exposure to NSAIDs, nonbiologic DMARDs, and glucocorticoids. Multiple mutations of the *MTHFR* gene have been identified, but their association with MTX toxicity is unclear. This case adds to a growing body of literature that could help inform the treatment of others in the future.

## 1. Introduction

Rheumatoid arthritis (RA) is a chronic inflammatory disorder affecting symmetric small and large joints associated with bone deformity and destruction. Common symptoms include pain, morning stiffness, and joint swelling. The clinical diagnosis is supported by elevated serum rheumatoid factor (RF) and anti-citrullinated peptide antibody (ACPA). Pulmonary, cardiovascular, renal, gastrointestinal (GI), and dermatologic involvement may be associated with disease progression or iatrogenically induced during medical management. RA treatment seeks to mitigate symptoms, prevent joint damage, and improve quality of life. Definitive treatment for rheumatoid arthritis begins with disease-modifying antirheumatic drugs (DMARDs) such as methotrexate (MTX). However, methotrexate has significant adverse side effects including pulmonary and hepatic toxicity. In addition, methotrexate treatment causes increased mucosal turnover and oral ulcerations which may increase the risk of GI complications [1]. We herein present a case where methotrexate may have been responsible for colonic perforation in a patient with RA.

## 2. Case

A 33-year-old nonsmoking Caucasian male with family history of RA and lupus, but no diverticulosis, presented to the clinic with complaints of symmetric hand, foot, neck, shoulder, knee, and ankle pain. He had morning stiffness and pain lasting several hours and denied ocular irritation, melena, skin rash, or history of sexually transmitted disease. He had some difficulty dressing himself including closing shirt buttons and tying shoes. He was diagnosed with polyarticular, RF positive, ACPA positive rheumatoid arthritis. He was started on methotrexate 17.5 mg orally weekly, folic acid, and prednisone. He used naproxen as needed. His symptoms did not improve, so he was started on sulfasalazine and hydroxychloroquine. Approximately eight months later, his arthritis was well controlled with methotrexate, low-dose oral prednisone (2 mg daily), and naproxen as needed; both sulfasalazine and hydroxychloroquine were discontinued.

**Figure 1:**
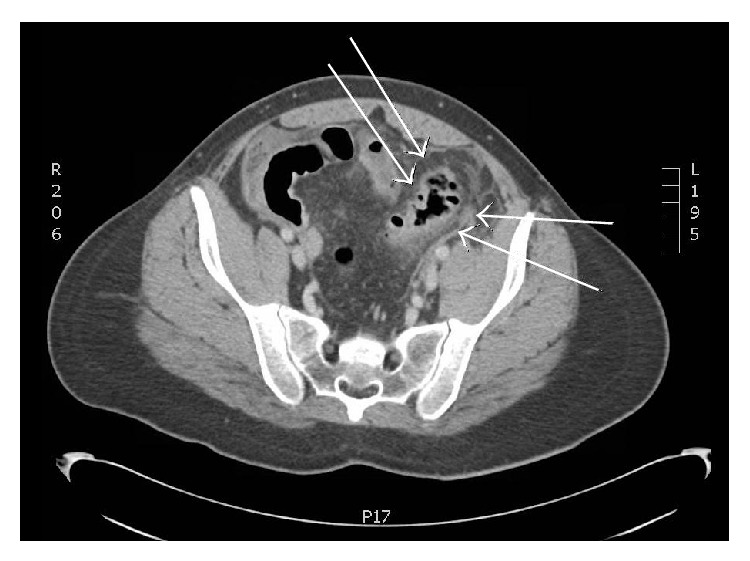
Wall thickening and inflammatory changes involving the sigmoid colon with multifocal pneumoperitoneum. Scattered hypodense collections in the abdomen and pelvis without obvious well-defined, rim-enhancing abscess.

One day he developed sudden, severe abdominal pain. X-rays in the emergency department identified mildly prominent loops of small bowel and a few scattered air-fluid levels, consistent with mild ileus. CT abdomen/pelvis without contrast showed perforated solitary sigmoid colonic diverticula with pneumoperitoneum; there was no abscess (Figure 1). He underwent urgent partial colectomy with diverting colostomy. Pathology revealed a lesion with hemorrhagic and granular mucosa, consistent with diverticulitis. There were no other diverticula. Over the next year, he underwent a colostomy take-down and reanastomosis with loop ileostomy and then finally ileostomy reversal. Additional testing revealed that he was heterozygous for the C677T allele methylenetetrahydrofolate reductase* (MTHFR)* gene mutation. He recovered well following his surgeries and has been off methotrexate since, declining to ever use it again. He subsequently did well on hydroxychloroquine and etanercept.

## 3. Discussion

Rheumatoid arthritis is associated with increased risk of upper and lower GI disease [2]. Although the incidence of upper GI disease, such as ulcers and bleeding, has decreased with newer therapeutic regimens, there has been no change in the incidence of lower GI complications (i.e., diverticulitis, colitis, and perforation).

Mpofu et al. showed that corticosteroids, independent of rheumatic diagnoses, are associated with increased risk of sigmoid diverticular abscess perforation (SDAP) [3]. Glucocorticoids and nonsteroidal anti-inflammatory drugs (NSAIDs) in combination further increase the risk of GI bleed and perforation in older patients with diverticulosis [4]. Gastrointestinal perforation is most frequent among patients treated with NSAIDs, nonbiologic DMARDs, and glucocorticoids. Prior studies have shown that exposure to DMARDs alone does not increase risk for bowel perforation [5]. Similarly, biologic agents generally do not increase the risk of perforation either. One exception is tocilizumab, a humanized anti-human interleukin-6 receptor antibody, which may cause intestinal perforation; it is contraindicated in older patients and patients with history of diverticulitis [5].

In this unusual case, our otherwise healthy young man on methotrexate, subphysiologic prednisone, and nondaily naproxen for RA suffered perforation of solitary, uninfected diverticula. His pathology was inconsistent with hereditary diverticulosis. The precise contributions of MTX, prednisone, and naproxen to his perforation are unclear, but a recent meta-analysis has suggested that polymorphisms of the* MTHFR* gene, such as the one our patient had, may contribute to methotrexate toxicity in patients with RA [6].

More investigation and clinical vigilance regarding the safety of methotrexate, NSAIDs, and corticosteroids alone or in combination will likely be beneficial. Furthermore, analysis of the potential association of* MTHFR* gene mutations (either heterozygous or homozygous) and methotrexate toxicity may prove to be crucial in preventing serious morbidity and mortality.
